# Methylhonokiol attenuates neuroinflammation: a role for cannabinoid receptors?

**DOI:** 10.1186/1742-2094-9-135

**Published:** 2012-06-20

**Authors:** Jürg Gertsch, Sharon Anavi-Goffer

**Affiliations:** 1 Institute of Biochemistry and Molecular Medicine, National Centre of Competence in Research NCCR TransCure, University of Bern, Bühlstrasse 28, Bern, CH-3012, Switzerland; 2 Departments of Behavioral Sciences and Molecular Biology, Ariel University Centre of Samaria, Ariel, 40700, Israel

**Keywords:** Alzheimer’s disease, Cannabinoids, CB_2_ receptors, Endocannabinoid System, Magnolia grandiflora, Medicinal plant, Methylhonokiol

## Abstract

The cannabinoid type-2 G protein-coupled (CB_2_) receptor is an emerging therapeutic target for pain management and immune system modulation. In a mouse model of Alzheimer’s disease (AD) the orally administered natural product 4′-O-methylhonokiol (MH) has been shown to prevent amyloidogenesis and progression of AD by inhibiting neuroinflammation. In this commentary we discuss an intriguing link between the recently found CB_2_ receptor-mediated molecular mechanisms of MH and its anti-inflammatory and protective effects in AD animal models. We argue that the novel cannabimimetic MH may exert its beneficial effects via modulation of CB_2_ receptors expressed in microglial cells and astrocytes. The recent findings provide further evidence for a potential role of CB_2_ receptors in the pathophysiology of AD, spurring target validation and drug discovery.

## Background

In a recent study published in *Journal of Neuroinflammation* Lee and colleagues report that the natural product 4′-O-methylhonokiol (MH) from *Magnolia grandiflora* L. potently inhibits lipopolysaccharide (LPS)-induced amyloidogenesis via anti-inflammatory mechanisms [1]. They have shown that chronic oral administration of 1 mg/kg of MH in mice strongly ameliorates LPS-induced memory impairment via inhibition of nuclear factor kappa B (NF-κB) and the gene expression of inducible nitric oxide synthase and cyclooxygenase-2. MH also inhibited the activation of astrocytes in the brain. The same group recently reported that MH attenuates the development of Alzheimer’s disease (AD) in Tg2576 mice [2], and inhibits different signaling cascades related to oxidative stress and mitogen-activated protein (MAP) kinases [3-5]. In the European patent application EP2327402A2 by Bioland Ltd. the authors report the invention of a method for treating or preventing amyloid-related diseases comprising administering a pharmaceutically effective dosage of MH or pharmaceutically acceptable salt thereof [6]. In this patent it is mentioned that MH inhibits acetylcholinesterase (AChE) and in a subsequent study it was shown that MH inhibits AChE activity at nM concentrations *in vitro*[7]. In yet another study by the same group, MH was shown to inhibit hydrogen peroxide and Aβ(1-42)-induced neurotoxicity in cultured neurons, as well as PC12 cells, by prevention of the reactive oxygen species generation and directly inhibited β-secretase activity and Aβ fibrilization *in vitro*[8]. Thus, MH could be a useful agent to prevent the neuroinflammation-associated pathogenesis or the progression of AD. However, beyond the AChE inhibition, none of the studies describe any molecular interaction of MH and its anti-inflammatory mechanism of action therefore remains elusive. In their article, Lee *et al*. suggested that inhibition of NF-κB and MAP kinases or the general antioxidative properties of MH are potential mechanisms by which this biphenyl natural product inhibits inflammation and amyloidogenesis [1]. However, from the data presented it is not clear whether the inhibition of signaling is a primary or secondary event, for example to receptor modulation. Moreover, some signaling effects were only observed at high nM or even μM concentrations *in vitro* which do not necessarily reflect the physiological concentrations in the brain. We therefore comment on a recently discovered molecular mechanism of action of MH that could well explain some of the anti-inflammatory effects observed.

### MH is a novel modulator of CB_2_ receptors and inflammation

In a previous study we have shown that MH is a potent and selective cannabinoid type-2 G protein-coupled (CB_2_) receptor ligand (*h*CB_2_*K*_i_ = 44 nM), triggering a novel type of heteroactive signaling (EC_50_ ~ 10 nM) [9]. In an *in vitro* profiling comprising more than 40 receptors MH was highly specific towards cannabinoid CB_2_ receptors at nM concentrations. Furthermore, MH did not interact with cannabinoid type-1 (CB_1_) receptors, which in the brain are predominantly expressed in neurons, and found in presynaptic sites of GABAergic and glutamatergic synapses where they in a retrograde manner inhibit the release of these neurotransmitters [10-12]. Whereas CB_1_ receptors mainly mediate the central side effects of cannabinoids, CB_2_ receptors are primarily associated with a broad range of inflammatory processes [13-16]. CB_2_ receptors are largely absent in the central nervous system (CNS) under normal conditions, but are upregulated in microglial cells and astrocytes under neuroinflammatory stimulation as it occurs in AD [17,18]. Indeed, CB_2_ receptors appear to mediate many of the anti-inflammatory actions of endocannabinoids, the arachidonic acid-derived lipids which non-specifically target cannabinoid receptors [19,20]. There is an overall agreement that endocannabinoids are released during oxidative and inflammatory stress and counterbalance inflammation by inducing a TH_1_-TH_2_ cytokine shift, although the exact mechanisms are not understood [14,21,22]. In our study we have shown that MH potently inhibits LPS-stimulated TNF-α expression and chemotaxis in macrophages in an apparently CB_2_ receptor-dependent manner, exerting anti-inflammatory and anti-osteoclastogenic effects [9].

### A role for CB_2_ receptors in the pathophysiology of AD

**Figure 1:**
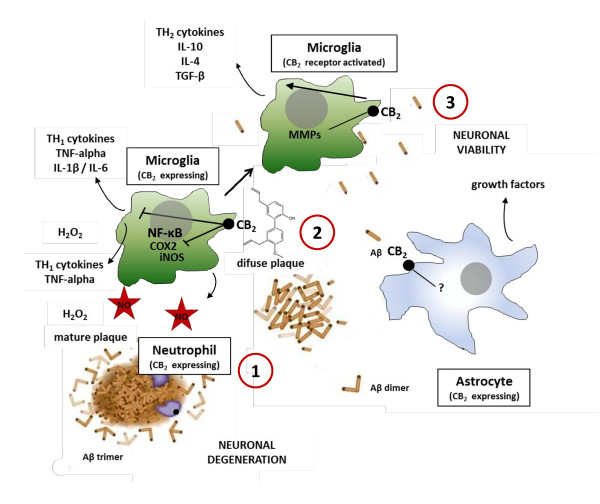
**Hypothetical model of how modulation of CB**_**2**_**receptors may attenuate inflammation in AD.** 1. Activated astrocytes, microglia, and macrophages release proinflammatory cytokines such as IL-1β, IL-6, and TNF-alpha in response to the deposition and accumulation of Aβ and the release of chemoattractants from damaged neurons. The endocannabinoid system counter regulates such processes and CB_2_ receptors seem fundamentally involved in this response. 2. MH modulates CB_2_ receptors, leading to inhibition of TH_1_ cytokines and possibly decreased chemotaxis 3. Decreased inflammation promotes Aβ phagocytosis (clearance) and neuronal viability.

The report by Lee *et al*. [1] is interesting because it links MH with the already established anti-inflammatory effects mediated via CB_2_ receptors in the brain. Since MH can act as both inverse agonist and agonist, depending on the specific signal pathway [9], it will be interesting to study the potentially positive and negative roles of CB_2_ receptor signal transduction in models of AD. A prominent effect of MH is the inhibition of macrophage migration induced by the endocannabinoid 2-arachidonoyl glycerol (2-AG), even though MH shows anti-inflammatory properties similar to 2-AG and other endocannabinoids [9]. Interestingly, the CB_2_ receptor mediates myeloid progenitor cell trafficking in the CNS, thus controlling inflammation in the brain [23]. The novel functionally heteroactive (dualistic) CB_2_ receptor ligand MH thus both inhibits and mimics the action of 2-AG via different pathways. Of note, 2-AG is the major arachidonic acid metabolite in the brain and a key lipid of the leukotriene network in the CNS [24]. It decreases brain edema, inflammation and infarct volume and improves clinical recovery via cannabinoid receptors [25,26]. Like 2-AG, MH can trigger calcium signaling in myeloid cells in a CB_2_ receptor-dependent manner [9]. Intriguingly, in this context, CB_2_ receptors have been directly associated with AD. It was shown that the activation of CB_2_ receptors stimulates *in situ* and *in vitro* β-amyloid removal by human macrophages [27]. Cannabinoids acting at CB_2_ receptors block β-amyloid-induced activation of cultured microglial cells and abrogate microglia-mediated neurotoxicity after β-amyloid addition to rat cortical co-cultures [28]. Furthermore, increased CB_2_ receptor expression was also found in neuritic plaque-associated astrocytes and microglia in brains from patients with AD [29]. Since endocannabinoids negatively regulate TNF-α, the downregulation via CB_2_ receptors may be a primary mechanism leading to inhibition of the downstream events including NF-κB activation, nitric oxide production and leukotriene synthesis [30,31]. Unfortunately, the role of endocannabinoids appears to be more complex because these promiscuous lipids also interact with other targets, such as peroxisome proliferator-activated receptors and the vanilloid receptor 1, which mediate the β-amyloid induced neuroinflammation in mice lacking the enzyme fatty acid amide hydrolase which regulates the metabolism of the endocannabinoid anandamide and other fatty acid ethanolamides [32]. Thus, agents selectively targeting CB_2_ receptors could be more advantageous to treat AD. We speculate that several of the downstream signaling effects of MH as reported by Lee *et al*. [1] are mediated via CB_2_ receptors. Along this line, the inhibition of Iκ-Bα phosphorylation in microglial cells by anandamide can be reversed by SR144528, a CB_2_ receptor-selective antagonist [33]. However, the synthetic cannabinoid WIN55212-2, a relatively potent non-selective CB_1_/CB_2_ receptor agonist, has been shown to inhibit NF-κB in neuronal cells in a receptor-independent manner, albeit at high concentrations [34]. Thus, MH may well inhibit NF-κB via CB_2_ receptors at nM concentrations, or at higher μM concentrations independently of receptor activation. CB_2_ receptor activation may change the cytokine pattern and shift the polarization of the microglia towards M2, thus reprogramming macrophages for β-amyloid removal (Figure 1). An obvious way to assess the involvement of CB_2_ receptors in the attenuation of neuroinflammation by MH would be to use CB_2_ receptor knockout mice. Alternatively, the effects of MH may be directly compared to the effects of honokiol, which is the biosynthetic precursor of MH that also targets kinases and NF-κB, exerting a range of anti-inflammatory effects *in vitro* at higher μM concentrations [35,36], but lacks the potent CB_2_ receptor affinity [9]. Both strategies might be used to assess the relative contributions of each action, namely the CB_2_ receptor modulation, general antioxidative effects or direct inhibition of kinases and NF-κB.

## Discussion

### The endocannabinoid system and neuroinflammation

Although AD is currently treated with cholinergic and glutamatergic therapies, which provide symptomatic benefit, the pathophysiology of AD is also widely associated with inflammation and aberrations of innate immunity [37]. Inflammation is not only involved in acute CNS conditions, such as stroke and traumatic injury, but it is also a central factor in chronic and neurodegenerative conditions such as AD, Parkinson’s disease and multiple sclerosis [38]. Nevertheless, the inflammation hypothesis of AD, as attractive as it appears, has not yet been corroborated in clinical trials. Recent attempts to treat AD with non-steroidal anti-inflammatory drugs and the TNF-α blocker entanercept were not successful [38,39], most probably due to the fundamental biochemical differences between neuroinflammation and peripheral inflammation [40]. However, novel pleiotropic anti-inflammatory mechanisms based on modulation of innate immunity, including the modulation of the endocannabinoid system, may be exploited. Because the CB_2_ receptor mediates different anti-inflammatory effects via multiple signaling pathways [22] it was previously suggested to be a drug target to treat neurodegenerative diseases [17,31]. However, to date only few preclinical studies have explored the pharmacological effects of the distinctly different CB_2_ receptor ligands (full agonists, partial agonists, inverse agonists, silent antagonists and protean agonists) in models of neuroinflammation and AD.

### CB_2_ receptor modulation by MH to target AD?

The promising preclinical results obtained with the novel CB_2_ receptor ligand MH may spur further research on the role of CB_2_ receptors in neuroinflammation in general and AD in particular. The findings reported by Lee *et al*. [1] are intriguing because they clearly indicate that MH is orally bioavailable to the CNS in mice, as well as active at relatively low doses. This is unexpected given the likely detoxification and phase II biotransformation of the biphenyl scaffold of this neolignan [41]. Thus, until the pharmacokinetics and metabolism of MH are studied it cannot be excluded that MH may potentially also act as a prodrug. Alternatively, MH crosses the blood–brain barrier and reaches the nM concentrations necessary to inhibit AChE and to modulate CB_2_ receptors, thus exerting a polypharmacological action on acetylcholine levels and inflammation. In addition to downregulating cyclooxygenase-2 gene expression [1], MH also directly inhibits COX1/2 [42], which may further contribute to its *in vivo* efficacy. MH is a relatively rare natural product of plant origin which is mainly found in the seeds of *M. grandiflora,* a tree native to Northern Mexico and the USA [43,44]. Its long use in traditional medicine and its mention in the United States Pharmacopoia as antimalarial and diaphoretic [44,45] suggest a lack of acute toxicity of MH, a major secondary metabolite in this medicinal plant.

## Conclusions

Because of the promising preclinical studies reported in the past, further studies are indicated to explore the therapeutic potential of CB_2_ receptor modulators such as MH and its CB_2_ receptor active derivatives [9] for AD drug discovery.

## Abbreviations

2-AG, 2-arachidonoyl glycerol; AChE, Acetylcholinesterase; AD, Alzheimer’s disease; CB1, Cannabinoid type-1 receptor; CB2, Cannabinoid type-2 G protein-coupled receptor; CNS, Central nervous system; GABA, Gamma-aminobutyric acid; LPS, Lipopolysaccharide; MAPK, Mitogen-activated protein kinases; MH, O-methylhonokiol; NF-κB, Nuclear factor kappa B; TNF-α, Tumor necrosis factor alpha.

## Competing interests

The authors declare no conflict of interests.

## Authors’ contributions

JG has written the commentary and drawn the figure. SAG has revised and complemented the commentary. Both authors read and approved the final manuscript.

## Authors’ information

JG is a professor and research PI at the Institute of Biochemistry and Molecular Medicine (IBMM), National Centre of Competence in Research NCCR TransCure, University of Bern, Switzerland. His research interests focus on the endocannabinoid system, molecular pharmacology and chemical biology, with an interest in natural products and drug discovery. SAG is a senior researcher and lecturer at the Departments of Behavioral Sciences and Molecular Biology, Ariel University Centre of Samaria, Israel. Her current research interests focus on the role of the endocannabinoid system in behavior and molecular pharmacology.
